# Urban–rural transportation accessibility: A novel geographical indicator for characterizing urban–rural integration

**DOI:** 10.1371/journal.pone.0343242

**Published:** 2026-02-26

**Authors:** Luming Liu, Zhenghong Liang, Lexuan Liu, Ruijing Qiao, Wangye Lu, Naixin Yin, Jiaxue Ji, Jie Li

**Affiliations:** 1 Guangxi Transportation Science and Technology Group Co., Ltd, Nanning, Guanxi, China; 2 Institute of International Rivers and Eco-security, Yunnan University, Kunming, Yunnan, China; 3 Ministry of Education Key Laboratory for Transboundary Eco-security of Southwest China, Yunnan University, Kunming, China; 4 Yunnan Key Laboratory of International Rivers and Transboundary Eco-security, Yunnan University, Kunming, Yunnan, China; 5 Zhenlai County Taxation Bureau, State Administration of Taxation, Baicheng, Jilin, China; Zhejiang Agriculture and Forestry University: Zhejiang A and F University, CHINA

## Abstract

The literature on urban–rural integration lacks not only a geographic indicator that incorporates geographical characteristics (e.g., location, topography, and road infrastructure) to characterize urban–rural integration but also methods of measuring the access costs of planning rural transportation infrastructure and individual village settlements (VSs). In response, our urban–rural transportation accessibility (URTA) model, using VSs as the unit of transportation accessibility, offers a new indicator to guide local transportation infrastructure planning and to capture the provincial-level heterogeneity of urban–rural access costs based on geographical characteristics. The model is constructed with data from over 140,000 VSs in the Chinese province of Yunnan and multilevel road network data for Yunnan from 2015 and 2023 based on GIS analysis. Moreover, because mountainous VSs may lack paved roads to connect to the road network, our model integrates results from the minimum cumulative resistance model to simulate least cost paths through the network. Using origin–destination analysis, we also calculated the travel time from each VS to its county center as a measure of URTA reflecting urban–rural integration. Among our findings, significant differences in travel time and path length between developed and impoverished counties reflect disparity in urban–rural integration. Longer travel times indicate less integration, and though times were consistently under 2.5 h in Guandu District, they ranged from 2 to more than 10 h in Gongshan County. Furthermore, on average, reaching county centers from the VSs took 2.13 h in 2015 and 1.46 h in 2023. Although URTA expanded in central Yunnan and improved considerably in the northwest, the southwest showed little change, which highlights significant disparity in urban–rural integration. Those results suggest that our method and indicator, by elucidating urban–rural integration in regions with high geographic spatial heterogeneity, can guide transportation planning in mountainous rural areas worldwide.

## 1. Introduction

Owing to their entrenched duality, urban and rural areas typically show pronounced disparity in terms of economic development, infrastructure, and public services [1–4], which has not only driven imbalances in economic growth but also spawned a range of secondary social problems [5–7].

Urban**–**rural integration, as an essential approach to promoting coordinated regional development, has become a sustained focus of scholarly research. In most studies, researchers have primarily adopted economic and social perspectives to construct multidimensional indicator systems to assess the level of integration. For example, some have examined the spatial configuration of urban and rural areas based on the distribution of population and enterprises [8], while others have analyzed the driving effects of the digital economy on urban–rural integration [9]. Beyond that, in their evaluation frameworks, several scholars have incorporated indicators related to quality of life [10], poverty alleviation outcomes [11], and overall regional development [12]. Although those contributions have provided valuable insights into the socioeconomic foundations of urban–rural integration, they have also often overlooked the profound influence of geographical characteristics on such integration.

A well-developed road network is not only a vital foundation for smooth socioeconomic connectivity but also a key indicator for evaluating the level of regional development. In turn, some scholars have sought to assess urban–rural integration from geographical perspectives. For instance, Avery et al. [13] have emphasized the importance of road network connectivity in enhancing urban–rural integration in order to achieve sustainable regional development. Lu et al. [14], meanwhile, have employed metrics of road network density to evaluate regional transportation connectivity and its implications for urban–rural spatial structure. Still, other scholars have examined the evolution of road networks and their role in shaping spatial linkages between urban and rural areas and highlighted the significance of transport infrastructure in advancing urban–rural integration [15–17]. Although approaches to date have advanced the spatial assessment of such integration, many continue to rely on simplified representations of spatial relationships. That limitation has reduced the accuracy of urban–rural integration assessments and impeded the quantification of practical challenges that geographical characteristic pose for urban–rural integration.

Distance, as a fundamental variable in geography [18], encompasses not only static spatial measures, such as Euclidean distance, which indicate the proximity between locations [19], but also travel-time-based accessibility, which more directly reflects the ease of inter-regional connectivity [20]. On that basis, many studies have focused on urban aspects to explore how transportation accessibility supports regional economic development and spatial organization. For example, the development of so-called one-hour economic circles has been shown to strengthen the influence of cities on surrounding areas by reducing travel times [21–23]. Transportation planning has also used improved transportation accessibility to promote the clustering of firms and optimize spatial layouts [24–27], while GIS-based measures have been applied to assess transport efficiency and regional development [28].

By contrast, rural areas are often widely dispersed across rugged, underdeveloped mountainous regions—examples include the Alps, Himalayas, Andes, Atlas Mountains, and Rocky Mountains—where obstacles to construction and low economic returns have made transportation infrastructure relatively weak and consequently exacerbated regional poverty and underdevelopment. Research focusing on rural aspects has thus primarily examined how improving transportation can promote rural development, including in terms of poverty reduction [29–31], gender disparity [32], public service delivery [33], and emergency response capacity [34]. Although a subset of studies emphasizing road network planning and infrastructure improvements have highlighted the importance of transportation accessibility from different aspects [35–37], most have examined only urban or only rural development. Moreover, few have established a direct connection between transportation accessibility and urban–rural integration or produced measures to characterize how spatial heterogeneity in transportation accessibility reflects disparity in levels of integration between urban and rural areas. That limitation poses problems for accurately assessing the spatial characteristics and heterogeneity of urban–rural integration. To address that gap in the literature, in our study we considered the topographic conditions in measuring urban–rural transportation accessibility (URTA) as a potential indicator for characterizing the degree of urban–rural integration.

Our study focused on the Chinese province of Yunnan, a region marked by rugged mountainous terrain with widely dispersed village settlements (VSs). With geographic characteristics representative of many mountainous areas worldwide, Yunnan is an ideal case for examining URTA and urban–rural integration. Using data from 2015 and 2023, we developed an URTA model to comprehensively measure transport accessibility by incorporating geographic location, topography, modes of transportation, and road conditions. The model calculates the travel time cost from each VS to its county administrative center in order to assess the level of transportation accessibility across all VS in the province. By comparing changes in URTA and their spatial distribution between 2015 and 2023, the model not only visually presents the difficulty of traveling from each VS to its county administrative center at the local scale but also characterizes spatial autocorrelation and heterogeneity among regions at the provincial scale, for a novel geographic indicator that can help to assess the level of urban–rural integration.

## 2. Materials and methods

### 2.1. Study area

The province of Yunnan, located in southwestern China (21°8’–29°15’ N, 97°31’–106°11’ E), has a resident population of 46.9 million and significant regional variation in its population distribution. According to the 2023 National Economic and Social Development Statistical Bulletin and the 2023 Yunnan Province Economic and Social Development Statistical Bulletin, 23.94 million people in Yunnan are urbanized, for an urbanization rate of 57.3%, which is nearly 8% less than China’s national average [38,39].

**Fig 1:**
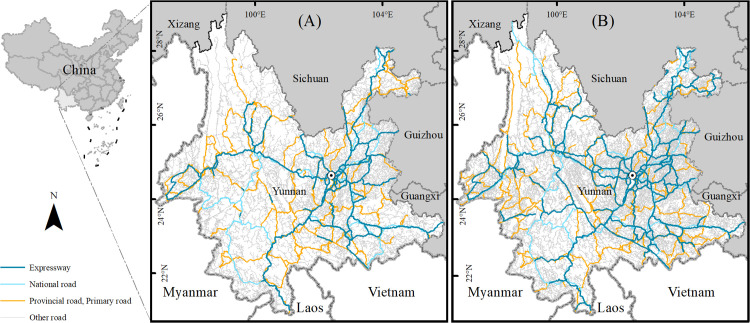
Differences in traffic in Yunnan, 2015 and 2023. (A) The road network in Yunnan in 2015 and (B) the road network in Yunnan in 2023. Road data were compiled from OpenStreetMap (https://www.openstreetmap.org/), Natural Earth (http://www.naturalearthdata.com/) and remote sensing interpretation. County boundary data were obtained from the National Geographic Information Public Service Platform (https://www.tianditu.gov.cn/). Map Review No.: GS (2024) No. 0650. All layers of the map were processed and visualized using ArcGIS 10.8, and no proprietary basemaps were used.

**Fig 2:**
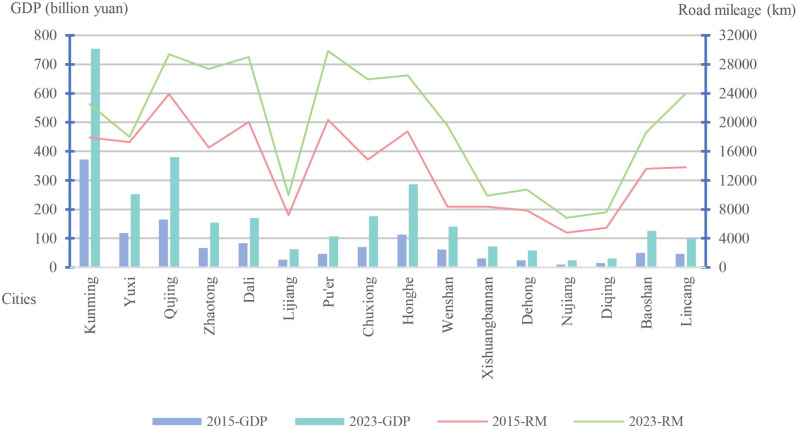
Road mileage (RM) and changes in GDP in cities and states in Yunnan 2015 and 2023.

Yunnan’s terrain is predominantly mountainous, with high peaks and deep valleys that differ in height by up to 6,664 m. Indeed, 43.54% of the province consists of steep slopes (>25°), which has induced significant differences in regional development. The unique characteristics of such mountainous environments, in significantly constraining the construction of transport infrastructure, pose a major challenge to the integrated development of urban and rural areas. For example, Fugong County and Gongshan County in the Nujiang Prefecture of northwestern Yunnan remain unconnected to any highways. Some VSs remain inaccessible by road, which severely impacts the everyday economic activities of residents there, while their host counties are also typically impoverished. In 2023, the GDP of Gongshan County, for instance, was 2.2 billion yuan, whereas the GDP of the eastern Guandu District was 147.4 billion yuan. Such significant regional economic disparity is closely linked to the province’s weak transport infrastructure. In recent years, Yunnan has made considerable efforts to develop its transportation system, and as the mileage of highways within the province has increased rapidly (Fig 1), the mileage of highways in cities and states has continued to increase (Fig 2), all of which has contributed greatly to the development of the regional economy. Therefore, analyzing the transportation accessibility from VSs to county centers is not only important for exploring practical strategies for integrated urban–rural development but also necessary to identify the level of economic development in VSs.

### 2.2. Datasets

All data used in our study were meticulously compiled and are presented in Table 1. Road networks were constructed using multiple sources of data and road sensing interpretation. All datasets employed are publicly available without copyright restrictions, and our use of them has complied fully with the terms and conditions set by the original data providers.

**Table 1:** The Datasets used in the study.

Dataset	Type	Temporal and spatial resolution	Sources	Use
Digital elevation models	Raster	2015, 2023 30 × 30 m	Geospatial Data Cloud Platform, ASTER Global Digital Elevation Model Version 3 (https://www.gscloud.cn/)	To calculate the resistance raster to simulate mountainous geomorphic features in the studied area
Village settlements (VSs)	Vector (points)	2015, 2023	Peking University Geographic Data Platform (https://geodata.pku.edu.cn/)	To determine the spatial location of VSs
Road networks	Vector (lines)	2015, 2023	1. Open Street Map (https://www.openstreetmap.org/) 2. Natural Earth (http://www.naturalearthdata.com/about/) 3. Remote sensing interpretation	To determine spatial locations for calculating travel times from VSs to county centers in a road network divided into (1) expressways, (2) national roads, (3) provincial and primary roads, (4) county roads, and (5) other roads
County borders	Vector (polygons)	2015, 2023	National Geographic Information Public Service Platform (https://www.tianditu.gov.cn/)	To determine the spatial locations of county borders
Yunnan Statistical Yearbook	Text	2015, 2023	Yunnan Provincial Bureau of Statistics (https://stats.yn.gov.cn)	To obtain social and economic indicators of the population

### 2.3. URTA model: Technical routes and model approaches

**Fig 3:**
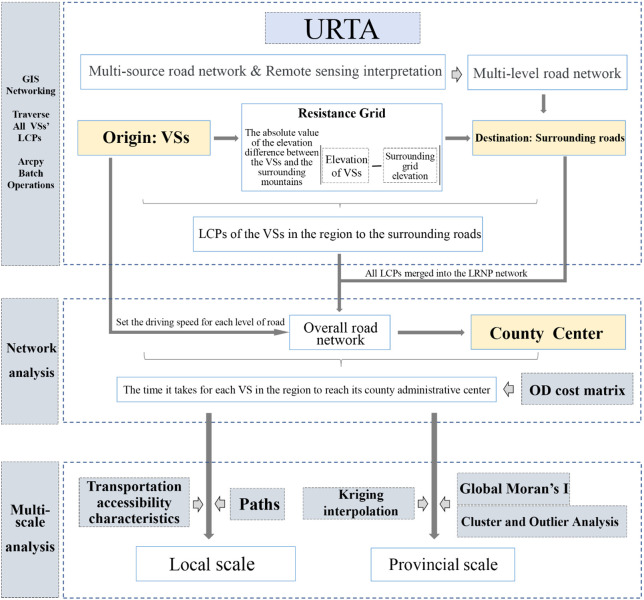
Flowchart of techniques used in developing the URTA model.

As shown in Fig 3, we developed an URTA model to assess URTA for all VSs in Yunnan. The model calculates the cost, in time, of traveling from each VS to its county center (i.e., typically the economic hub) and analyzes the spatial distribution of those data and their changes over time.

Developing the URTA model involved three steps. First, to form a basic road network, road data from multiple sources were analyzed using GIS. Remote sensing interpretation was employed to identify missing rural paths, which were subsequently integrated into the multilevel road network via GIS. Second, least cost paths (LCPs) were calculated to simulate the walking paths of rural residents from their home VSs to the road. Those paths were also integrated into the road network in order to form a comprehensive road system. Third, least road network paths for rural residents to the county center were identified. Using the Network Analysis tool set in ArcGIS 10.8, different travel speeds were assigned to roads of various levels, and the origin–destination cost matrix algorithm was applied to calculate the most time-efficient routes. After completing those three steps, we obtained a dataset for the URTA model that detailed the travel times from all VSs in Yunnan to their county centers.

Last, the research results were characterized on two scales: the local scale, in which the travel time and route from each VS to the county center were analyzed using the mentioned dataset; and the provincial scale, in which spatial autocorrelation and regional heterogeneity were examined using Kriging interpolation, Global Moran’s I, and cold and hot spot analysis.

### 2.4. URTA model: Implementation

#### 2.4.1. Multilevel road network.

Because the data about the road network from a single source might have been incomplete, we combined data from sources such as Open Street Map and Natural Earth on ArcGIS 10.8 to create a multisource road network. Missing rural paths and highways were filled in and supplemented using remote sensing interpretation, which yielded a comprehensive multilevel road network by maximizing the inclusion of all relevant roads. During preprocessing, pedestrian-only routes (e.g., footpaths and hiking trails) were excluded from the dataset in order to uphold the assumption of vehicular travel. That exclusion, applied by collecting data for motorable roads only, precluded the need for post hoc adjustments. The approach also minimized bias arising from mixed path types and ensured accessibility estimates that were relatively reliable and comparable.

#### 2.4.2. Paths from VSs to the nearest Road.

Due to complex mountainous terrain in Yunnan, not all VSs are accessible by vehicles. Studies have shown, however, that constructing a resistance surface based on the absolute elevation difference between each VS and its surrounding terrain effectively captures travel obstacles in such areas [40]. Following that approach, we generated a resistance raster grid at a 30 m resolution derived from the absolute elevation difference between the VSs and their surrounding terrain, all based on data from digital elevation models. The Cost Distance tool in ArcGIS 10.8 was subsequently applied to simulate the LCP from each VS to the nearest road. To efficiently process the LCP calculations for 145,120 VSs, we implemented an automated loop using the ArcPy library in ArcGIS 10.8.

#### 2.4.3. Integrated road network.

The LCPs from each VS to the nearest road were merged with the road network, with values assigned based on speed limits. A network dataset was created that followed topological rules to correct access logic and topological errors (e.g., expressways not intersecting with regular roads and overlapping roads). The result was a unified road network combining pedestrian LCPs and existing roads.

#### 2.4.4. URTA calculations.

Once rural residents reach the road network, they are assumed to need to travel to the county center using available transportation. To determine the shortest path from each VS to the county center, we used the Network Analysis tool set in ArcGIS 10.8, which accounted for the actual road network’s layout, types of roads, and traffic conditions. After setting the maximum speed limit for each road class in the network dataset (see Table 2), we created an origin–destination cost matrix (see S2 Table). The locations of each VS and its county center were added as the origin and destination points, respectively, to calculate the shortest path and travel time. Travel time was estimated based on road-class-specific speed limits and does not incorporate short-term traffic congestion that varies by time or day.

**Table 2:** Maximum speed on each Road class (km/h).

Road class	Max speed (km/h)	Description
Expressway	120	Controlled-access highways for high-speed traffic
National road	80	Major national trunk roads connecting large cities
Provincial road, primary road	60	Regional highways or arterial roads
County road	40	County-level roads, varying in width and condition
Others road	30	Village roads or unclassified local routes
Least cost path (i.e., LCP)	5	The path from a village settlement to its nearest road

### 2.5. Spatial characteristics of local URTA

Using the above-described method, we calculated the travel time for each VS in Yunnan. The Network Analyst extension for ArcGIS 10.8 was used to pinpoint the optimal path from each VS to the county center and highlighted the path and gap in travel times on a local scale.

### 2.6. Characteristics of URTA in Yunnan

#### 2.6.1. Patterns of URTA in Yunnan.

Using the Geostatistical module in ArcGIS 10.8, we set the total travel time calculated earlier, which represent the time cost of traveling from VSs in Yunnan to county centers, as the base parameter. Ordinary Kriging interpolation was performed to derive patterns of URTA in Yunnan.

#### 2.6.2. Spatial autocorrelation features and characteristics of regional heterogeneity.

Due to significant geospatial heterogeneity in Yunnan, we employed Global and Local Moran’s I spatial statistical methods to characterize the spatial and temporal heterogeneity of URTA in Yunnan for 2015 and 2023. Considering the county as the fundamental unit for policymaking and acknowledging the impact of the geological and topographical features of Yunnan on transportation infrastructure, we employed Open Geoda to extract the mean URTA for each county and district. Global Moran’s I was calculated to analyze the spatial autocorrelation of URTA in each county. Moreover, cold and hot spot analysis (i.e., using Getis–Ord Gi*) and cluster and outlier analysis (i.e., using Anselin Local Moran’s I) in the Spatial Statistics module of ArcGIS 10.8 were performed to identify cold and hot spots, Local Moran’s I, and URTA in anomalous counties across Yunnan.

#### 2.6.3. Spatial regression analysis.

Building on the mentioned spatial URTA measurements, we employed spatial econometric models to quantitatively assess the impact of URTA on regional economic development at the county level in Yunnan for 2015 and 2023. The dependent variable was per capita income, with the core explanatory variable being the mean county-level URTA index. Because the original URTA values represented travel time, with higher values indicating poorer URTA, the index was transformed by taking its negative to ensure a positive association with URTA. In the model, to control for potential confounding socioeconomic factors, we included mean wage, total retail sales, enrollment in general secondary schools, and outputs of the primary, secondary, and tertiary industries as control variables. All economic variables were log-transformed to mitigate skewness and heteroscedasticity, and independent variables were standardized to eliminate scale effects and enhance the comparability of the estimates. Based on county-specific geographic coordinates, an eight-nearest neighbor spatial weight matrix with row standardization was constructed to capture spatial adjacency. Initial ordinary least squares (OLS) regressions were conducted, followed by Moran’s I tests on residuals to detect spatial autocorrelation. Given the presence of significant spatial dependence, spatial error models, spatial lag models, and spatial Durbin models were subsequently estimated to capture various forms of spatial effects. Variance inflation factor (VIF) diagnostics were also applied to assess multicollinearity, which ensured robust model estimates. That methodological framework provided a rigorous spatial econometric basis for analyzing the influence of URTA on county-level economic outcomes in Yunnan.

#### 2.6.4. Heatmap analysis of URTA and GDP.

Spatial visualization was undertaken to illustrate changes in URTA and per capita GDP at the county level in Yunnan between 2015 and 2023. The differences in URTA and income were calculated by subtracting the 2015 values from the 2023 values for each county, the results of which were subsequently mapped on heatmaps using ArcGIS Pro 3.0. A consistent color gradient scheme was applied to visually distinguish areas of improvement (i.e., in warm colors) and decline or minimal change (i.e., in cool colors). The heatmaps were intended to provide an intuitive spatial contrast of gains and losses in URTA alongside patterns of economic growth. Per capita GDP data were sourced from official statistical yearbooks, and all spatial data were aligned to the same coordinate system (i.e., WGS 1984). The spatial overlay and visualization helped to reinforce the observed relationship between improvements in URTA and socioeconomic development.

### 2.7. Sensitivity analysis

Due to the large volume of data, 256 VSs in Gongshan County were selected as representatives to assess the sensitivity of the URTA model to assumed travel speeds. Because all road types except LCPs have clearly defined speed limits and stable parameters, we focused exclusively on varying the travel speed on the LCPs. The baseline travel speed for LCPs was set to 5 km/h, which represented pedestrian walking speed, while alternative speeds of 10 km/h and 15 km/h were used to simulate potential travel using farm vehicles and electric bicycles, respectively. Based on those scenarios, a sensitivity index was calculated to evaluate the model’s stability. For each tested speed $X_{i}$, the corresponding travel time $T_{i}$ was calculated. Using the baseline speed $X_{i}\;$= 5 km/h and its associated travel time $T_{i}$, the index was computed as follows [41]:


$$X_{i}=\frac{(T_{i}-T_{\text{0}})/T_{\text{0}}}{\left(X_{i}-X_{\text{0}}\right)/\;X_{\text{0}}}$$ (1)

in which $S_{i}$ quantifies the relative responsiveness of travel time to changes in speed. A negative $S_{i}$ indicates a decrease in travel time with increased speed. Values of $\left|S_{\dot{i}}\right|$ < 1 suggest a non-significant sensitivity of travel time to variations in speed. The index provides a basis for evaluating the robustness and applicability of the model under different travel conditions.

## 3. Results

### 3.1. Local scale

**Fig 4:**
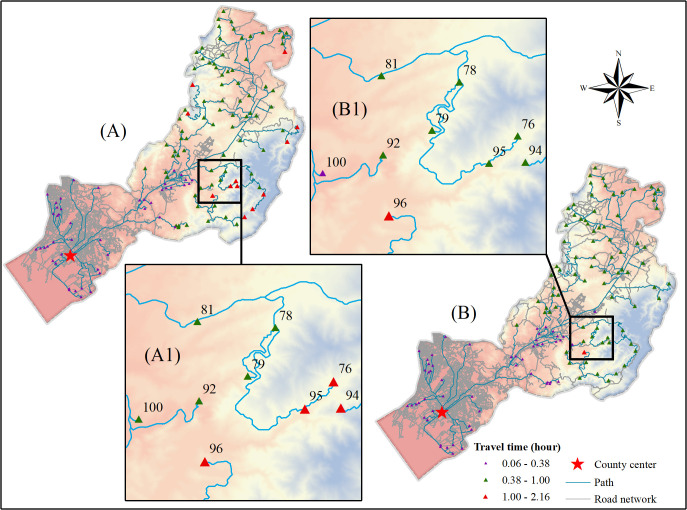
Paths and travel times from VSs to the county center in Guandu District, 2015 and 2023. (A) Travel paths and times from VSs to the county center in Guandu District in 2015 and (B) travel routes and times from VSs to the county center in Guandu District in 2023, with (A1) and (B1) as enlarged views of the areas in (A) and (B) that emphasize the specific travel time reductions in 76, 94, 95, and 100. The background uses red to represent low altitudes and blue to represent high altitudes. Elevation background from ASTER GDEM v3 (NASA/METI, https://www.gscloud.cn/). Road data come from OpenStreetMap (https://www.openstreetmap.org/), Natural Earth (http://www.naturalearthdata.com/), and remote sensing interpretations; VSs come from Peking University’s Geographic Data Platform (https://geodata.pku.edu.cn/); and county borders come from the National Geographic Information Public Service Platform (https://www.tianditu.gov.cn/). Map Review No.: GS (2024) No. 0650. Maps were created by the authors using ArcGIS; no proprietary basemaps were used.

Due to the large volume of data, including basic statistical values of the number of municipal-level VSs in Yunnan and travel time to the county center (see S1 Table), we selected two representative regions to demonstrate the spatial characteristics of URTA at a local scale: Guandu District in central Yunnan, Kunming, and Gongshan County in northwest Yunnan, Nujiang. As shown in Fig 4, Guandu District, a subdistrict of Kunming, has travel times within 2.5 h, and with few exceptions, travel from VSs to the county center takes less than 1 h. The LCPs for those VSs are relatively short, and most are located near roads, which ensures easy access to transportation. Those trends suggest that more developed areas have relatively well-established transportation infrastructure, which contributes to higher URTA levels. Furthermore, as Fig 4 shows, total travel time declined from 2015 to 2023. As shown in Fig 4A1 and 4B1 in particular, the travel times for 76, 94, 95, and 100 decreased significantly.

**Fig 5:**
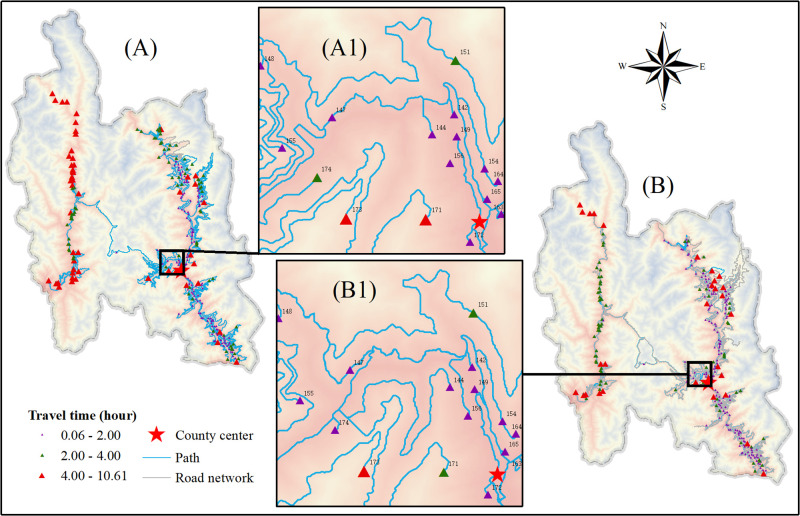
Paths and travel times from VSs to the county center in Gongshan County in 2015 and 2023. (A) Travel paths and times from VSs to the county center in Gongshan County in 2015 and (B) travel routes and times from VSs to the county center in Gongshan County in 2023, with (A1) and (B1) as enlarged views of the areas in (A) and (B) that emphasize the specific travel time reductions in 171 and 174. The background uses red to represent low altitudes and blue to represent high altitudes. Elevation background comes from ASTER GDEM v3 (NASA/METI, https://www.gscloud.cn/); road data come from OpenStreetMap (https://www.openstreetmap.org/), Natural Earth (http://www.naturalearthdata.com/), and remote sensing interpretation; VSs come from Peking University’s Geographic Data Platform (https://geodata.pku.edu.cn/); and county borders come from National Geographic Information Public Service Platform (https://www.tianditu.gov.cn/). Map Review No.: GS (2024) No. 0650. Maps were created by the authors using ArcGIS; no proprietary basemaps were used.

By contrast, as shown in Fig 5, Gongshan County, as a typical poverty-stricken county in Yunnan, had travel times from VSs to the county center ranging mostly from 2 to 4 h, although some exceeded 10 h, which makes transportation highly inconvenient. Because the LCPs from VSs to the nearest road were especially long, lower URTA emerged. Moreover, the region has few roads to begin with, and some VSs have remained unconnected to any road network, which causes significant travel difficulties for residents and hinders urban–rural integration. Travel times to the county center in 2023 decreased considerably compared with 2015, as illustrated in Fig 5 (A1 and B1), with 171 and 174 showing particularly substantial reductions in travel time. The data also reveal that Gongshan County had a GDP of 715 million yuan in 2015 but a GDP of 2.2 billion yuan in 2023, for a significant 200% increase over the period.

### 3.2. Provincial scale

#### 3.2.1. Patterns of URTA in Yunnan.

**Fig 6:**
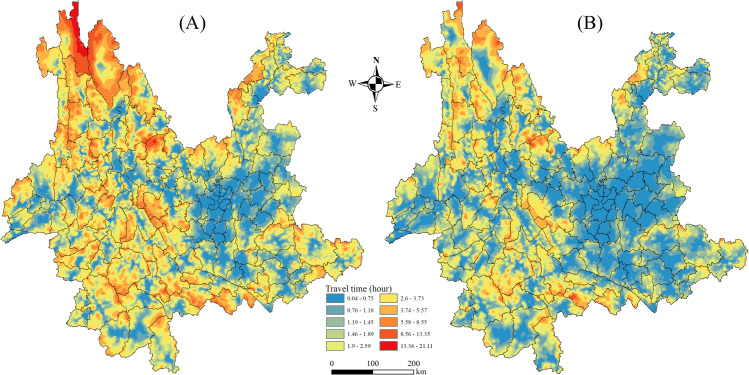
Patterns of URTA in Yunnan. (A) URTA in 2015 and (B) URTA in 2023. The black borders represent the borders of the 129 counties in Yunnan, as shown in S1 Fig. County borders come from National Geographic Information Public Service Platform (https://www.tianditu.gov.cn/). Map Review No.: GS (2024) No. 0650. Maps were created by the authors using ArcGIS; no proprietary basemaps were used.

In 2015, as shown in Fig 6A, although the longest travel time from a VS in Yunnan to its county center was 25.13 hours, the mean time was 2.13 hours (*SD* = 1.83). Moving outward, the URTA gradually worsened in irregular concentric rings centered around the central Yunnan region, including Kunming, Yuxi, and Qujing, with the lowest point located near Baima Snow Mountain in Deqin County, Diqing Prefecture. The eastern region had better URTA than the western region, and the central region outperformed the northern and southern regions. Northwestern Yunnan, including Diqing, Lijiang, and Nujiang, as well as southwestern Yunnan, including Linxiang, Pu’er, and the southern part of Honghe, had poorer URTA. By contrast, the southern regions of Kunming, including Wuhua District, Panlong District, Yuxi, and Qujing, which constitute the province’s economic core, especially the provincial capital of Kunming, boast well-developed infrastructure, a concentrated population, and a favorable geographic location, all of which make it a key area for socioeconomic connectivity.

As shown in Fig 6B, in 2023 the longest travel time from VSs in the province to the county center was 19.28 hours, though the mean time was 1.46 hours (*SD* = 1.24). Compared with 2015, the gap in URTA between VSs gradually decreased. The radiating effect of central Yunnan became more prominent, with regions showing a transportation advantage forming an east–west linear distribution. URTA in Dehong and Baoshan in western Yunnan, as well as Linxiang in southwestern Yunnan, improved as well, with notable progress in Diqing and Nujiang in northwestern Yunnan.

#### 3.2.2. Spatial autocorrelation features and characteristics of regional heterogeneity.

Moran’s index is used to quantify the first law of geography. Whereas Global Moran’s I reflects the overall spatial clustering and spillover effects of a region, Local Moran’s I emphasizes the geographical correlation between local units. The mean URTA for each county in the province was calculated, and Global Moran’s I was computed. Global Moran’s I was 0.43 in 2015 (*p* = 0.001, *z* = 9.0) and increased to 0.49 in 2023 (*p* = 0.001, *z* = 8.85), as shown in S2 and S3 Figs. The results also indicate significant positive spatial autocorrelation in the mean URTA across counties. The scatterplot of Global Moran’s I values demonstrates that most sample points are located in the first and third quadrants, which suggests a positive spillover effect with high–high and low–low clustering patterns. As a carrier of spatial spillover, the similarity in natural geography facilitated the positive spatial correlation of URTA across counties.

**Fig 7:**
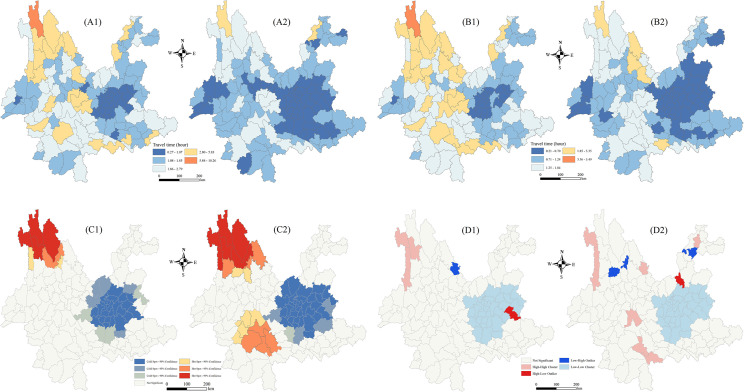
Regionally heterogeneous characteristics and Local Moran’s I of URTA in counties in Yunnan. (A) Means, (B) standard deviations, (C) hot spots and cold spots, and (D) Local Moran’s I. For each figure, the subfigure labeled “1” (e.g., A1, B1, C1, and D1) represents the URTA statistical characteristics in 2015, while the subfigure labeled “2” (e.g., A2, B2, C2, and D2) represents the URTA statistical characteristics in 2023. County borders come from the National Geographic Information Public Service Platform (https://www.tianditu.gov.cn/). Map Review No.: GS (2024) No. 0650. Maps were created by the authors using ArcGIS; no proprietary basemaps were used.

The spatial statistical characteristics of URTA at the county level were analyzed using natural breaks classification and by dividing the means and standard deviations into five categories (Fig. 7A1). In 2015, western Yunnan had poor URTA compared with eastern Yunnan, with the northwestern and southwestern areas being especially underdeveloped. Deqin, for example, had a mean travel time exceeding 10 hours, while areas such as Diqing and Nujiang also faced challenges. By contrast, eastern Yunnan, except for some regions in Zhaotong and Honghe, exhibited higher levels of URTA. As shown in Fig 7A2, there was noticeable improvement by 2023, and the lowest URTA category even disappeared. The northwestern and southwestern regions experienced significant gains, although the overall trend in distribution remained largely unchanged, with Deqin still having the worst URTA. The standard deviation reflects the degree of dispersion, and its distribution mirrors that of the mean; areas with lower URTA also had higher standard deviations, and vice versa. The distribution of mean URTA values and standard deviations was consistent (Fig 7B1 and 7B2). Recent improvements in transportation infrastructure notably enhanced URTA in the southwest and northwest, though the relative development levels remained unchanged and continued to align with the east–west disparity in URTA.

Using ArcGIS 10.8 for local spatial autocorrelation analysis, Local Indicators of Spatial Association (LISA) cluster maps were generated. As shown in Fig. 7C1, seven counties in northwestern Yunnan in 2015 (e.g., Diqing, Nujiang, and Lijiang) were identified as hot spots with high travel times and thus represented the regions with the lowest URTA in Yunnan. The low-value areas were concentrated in a ring of 31 counties in central Yunnan, including Chuxiong, Kunming, Qujing, and Yuxi. Those regions, being relatively developed urban areas for Yunnan, were characterized by dense road networks. As shown in Fig 7C2, compared with 2015, the cold and hot spots expanded in 2023. The number of counties with longer travel times in the northwest increased to 12, and the cold spots in central Yunnan also expanded to include Luoping and Qiubei. In the northern part of Pu’er and eastern Lincang, eight counties emerged as new low-URTA hotspots, thereby highlighting a more prominent regional clustering effect. As shown in Fig. 7D1, in 2015 Local Moran’s I not only indicated high–high clusters in the northwest and low–low clusters in the southeast but also identified some counties with high–low spatial outliers, including Wuding and Shuangbai in Chuxiong, Luquan in Kunming, Qiubei in Wenshan, and Shizong in Qujing. Those areas demonstrated poorer URTA than their neighboring regions, which indicates significant spatial heterogeneity. As shown in Fig. 7D2, in 2023 compared with 2015, the high–high cluster areas increased in Pu’er, whereas the high–low outliers no longer included Shizong in Qujing. New low–high clusters appeared, including the ancient town of Lijiang and Jianchuan in Dali, where URTA was better than in the surrounding regions.

Because all counties showed improvements in mean URTA, newly emerging cold spots and hot spots, including in Pu’er and certain counties in Lijiang and Nujiang in northwestern Yunnan, were likely due to slower improvements in URTA than in neighboring regions. The increase in cold spots in central Yunnan occurred due to substantial improvements in URTA in the area. High–low and low–high clusters appeared around those cold and hot spots, where the disparity in URTA between the hot spots and their surrounding regions was particularly significant.

#### 3.2.3. Spatial regression analysis.

The results of spatial regression indicated that residual Global Moran’s I values of the OLS models in 2015 and 2023 were 0.262 (*p* < 0.01) and 0.484 (*p* < 0.01), respectively, which demonstrate significant spatial autocorrelation in county-level incomes and suggest that the OLS models failed to fully capture spatial effects. The variance inflation factor (VIF) test, meanwhile, showed that the maximum VIF was less than 5, which indicates no severe multicollinearity and ensures the reliability of the model estimates. When spatial econometric models (i.e., Spatial Error Model, Spatial Lag Model, and Spatial Durbin Model) were employed, URTA still exhibited a significant positive impact on per capita income. On that count, the coefficients in spatial models were 0.026–0.031 (*p* < 0.1) in 2015 and 0.016–0.018 (*p* < 0.1) in 2023, which confirm the promoting effect of improved URTA on regional income growth, as shown in S3 Table.

#### 3.3.4. Heatmap analysis of URTA and GDP.

**Fig 8:**
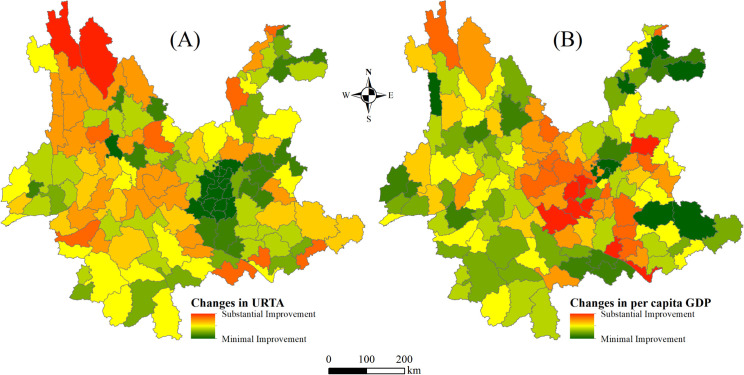
Heatmaps of changes in URTA and per capita GDP, 2015–2023. (A) Heatmap of changes in URTA and (B) heatmap of changes in per capita GDP. Color gradients from red to green represent the magnitude of change in each variable, with red indicating substantial improvement or growth and green denoting minimal improvement or near-zero change. Both panels visualize spatial trends using continuous heatmaps at the county level. County borders come from National Geographic Information Public Service Platform (https://www.tianditu.gov.cn/). Map Review No.: GS (2024) No. 0650. Maps were created by the authors using ArcGIS; no proprietary basemaps were used.

To complement the regression analysis, Fig 8 presents heatmaps depicting the spatial distribution of changes in URTA and per capita GDP at the county level between 2015 and 2023. Panel (A) shows that counties in the northern and southern border regions experienced the most substantial improvements in URTA (in red), while central areas saw relatively modest changes (in green). By contrast, Panel (B) illustrates that income growth was most prominent in central and southeastern counties, with several border counties showing minimal gains. The contrasting spatial patterns in the two heatmaps underscore dynamics of uneven regional development and support the model-based conclusion that improvements in URTA have had a spatially heterogeneous but generally positive effect on income growth.

### 3.3. Sensitivity analysis

The sensitivity analysis demonstrated that travel time decreased in response to increases in path speed parameters from 5 km/h to 10 km/h and 15 km/h. The median sensitivity indices were −0.12 and −0.08, with corresponding means of −0.15 (*SD* = 0.14) and −0.10 (*SD* = 0.09). Those results indicate that URTA improves as speed increases, as shown in S4 Fig. The sensitivity indices exhibit a tight distribution without extreme outliers, for a pattern that reflects good model stability across variations in speed parameters.

## 4. Discussion

### 4.1. Applicability of the URTA model

In the context of rapid urbanization, urban–rural integration has become a crucial issue for coordinated regional development [42–44]. Our study addressed the shortcomings of existing evaluation systems, which often overlook the complexity of transportation conditions and urban–rural connectivity, by introducing the novel geographic indicator of urban–rural transportation accessibility—for short, URTA. By precisely measuring the travel distance and time from each VS in Yunnan to county centers, we established a URTA indicator system to assess the balance of regional development and the degree of urban–rural integration.

Unlike traditional studies that have predominantly relied on single indicators such as Euclidean distance or road density [45–49], which often fail to capture the multifaceted nature of URTA, our research followed a more holistic approach. By integrating multiple conditions such as topography, transport modes, and roads, our study overcame limitations identified in previous research. For instance, Lei et al. [45] explored the role of transit hubs but did not fully incorporate regional spatial heterogeneity, while Zhaoying and Yilin focused on broad quantitative measures of urban–rural integration [49]. We placed particular emphasis on the development of transportation in mountainous regions, which have often been neglected in conventional analyses. Using Yunnan as a case study, we not only identified spatial imbalances in urban–rural transportation development but also examined their impact on urban–rural integration. Those insights contribute to current understandings of how inequities in transportation hinder the development of more integrated urban–rural systems. Furthermore, the findings provide a solid theoretical foundation for improving infrastructure planning and policy development, especially in areas with complex topographies, where transportation barriers are a significant challenge to achieving balanced urban–rural development.

Because our study also innovatively incorporated urban–rural integration into the analysis of URTA, it validated the URTA indicator as a reliable measure of integration. That approach, which goes beyond conventional measures, provides a more nuanced understanding of the spatial dynamics between urban and rural areas. Although applied to Yunnan only, the methodology possesses broad applicability for regions with complex topographies. In particular, it offers valuable insights for transportation planning and policymaking, especially by identifying terrain-induced barriers to urban–rural integration. By highlighting spatial inequalities in URTA, our findings support more targeted, more efficient resource allocation that can ensure that the development of infrastructure addresses the unique challenges faced by mountainous and/or geographically isolated regions.

### 4.2. Targeted interventions

Our study demonstrated that reductions in travel costs and improvements in URTA have significantly facilitated urban–rural integration and, in turn, fostered a virtuous cycle of regional economic and social development (Figs 6 and 7). However, spatial statistical analyses revealed persistent disparity, with northwestern counties such as Diqing and Nujiang continuing to be characterized by low URTA and limited infrastructural improvements despite recent investments. Those areas have also exhibited high–high clusters of poor URTA (Fig 7C2), which underscore persistent spatial agglomerations of URTA deficiencies that hinder the efficient flow of production factors and impede deeper urban–rural integration.

To address those challenges, we propose a set of differentiated, targeted interventions aligned with the spatial heterogeneity identified in our study. First, priority should be given to phased upgrades and the maintenance of trunk roads in severely inaccessible counties, particularly Diqing, Fugong, and Nujiang. As demonstrated in our analysis, a substantial proportion of VSs in those areas still lack direct road access. Targeted investments to achieve universal VS-to-road connectivity would markedly reduce travel times to county centers and significantly improve URTA. Second, given the persistently long travel times and low URTA values for VSs in specific regions of Yunnan, particularly in the northwest and southwest, a targeted VSs relocation strategy should be considered (Fig 6). For VSs in areas such as Deqin County and parts of Nujiang Prefecture, severe geographical and infrastructural constraints limit connectivity. Relocating entire VSs to sites closer to county centers or transport nodes could reduce travel costs and improve access to basic services. Third, as Figs 1 and 6 clearly demonstrate, the expansion of expressways between 2015 and 2023 substantially improved county-level accessibility across Yunnan. That finding is consistent with the results of Chen et al., who identified expressway expansion as a key driver of improved regional accessibility [50]. Therefore, we recommend further extending the provincial expressway network with the goal of achieving full expressway coverage to all counties. Along with expanding total expressway mileage, planning should prioritize the integration of expressways with regional arterial roads and county-level feeder networks to maximize gains in URTA.

Overall, those targeted interventions should not only help to reduce spatial heterogeneity in transport URTA but also provide more stable, more efficient channels for the movement of people and resources. Together, they can establish both institutional and infrastructural foundations to accelerate urban–rural integration and promote balanced regional development.

### 4.3. Limitations

We encountered several challenges and limitations while analyzing URTA in Yunnan. First, when using ArcPy on ArcGIS 10.8 to calculate the LCPs for VSs, we experienced certain computational failures due to program limitations and model imperfections. Those failures affected a small number of VSs, and given their minimal impact on the overall results, they were not considered in the final analysis.

Second, data collection posed significant challenges, particularly with respect to rural roads and footpaths in mountainous areas that are accessible to pedestrians only. Those roads are often not formally recorded and/or difficult to locate within existing geographic information databases. As a consequence, those types of roads were excluded in the analysis. Although that exclusion may have caused some discrepancies in the travel times computed for certain VSs, because those roads are relatively few and infrequently used, their impact on the overall findings is negligible.

Third, due to the lack of field survey data and crowdsourced mobility data (e.g., GPS traces from local transport providers), our estimates of URTA could not be independently validated through real-world observations of transportation in action. Although we acknowledge the uncertainty associated with potential unmapped paths and addressed it by selecting and integrating multiple data sources to maximize accuracy, such uncertainty is also inherent and cannot be completely eliminated.

In addition, travel time was calculated with respect to the county-level administrative center associated with each village settlement. In some cases, a village settlement may be geographically closer to the county center of a neighboring county, which was not considered in the present analysis.

Last, we did not incorporate the railway network into the overall road network analysis. That decision was made because our focus was on URTA within individual counties. Railway stations are typically situated at the county level, and their influence is more pronounced in enhancing intercounty and interprovincial URTA, with a minimal effect on the URTA of VSs to county centers.

In sum, although those limitations were present, their impact on the overall findings was minimal and did not substantively affect the conclusions drawn. The study’s robustness and reliability were ensured in light of those considerations. Future research could aim to optimize ArcGIS tools or employ alternative methods to improve the accuracy of LCP calculations, enhance the collection and analysis of data on rural roads (e.g., through field surveys or new technologies), consider incorporating the railway network, and explore alternative destination choices beyond administratively affiliated county centers to better reflect residents’ actual travel behavior.

## 5. Conclusion

We developed an URTA model that integrates multiple sources of data as well as conducted remote sensing interpretation, which together overcome the gaps in data typically caused by relying on a single data source. By combining methods to calculate LCPs and least road network paths, the study comprehensively accounted for conditions such as terrain, roads, and modes of transportation and effectively represented impedances to travel in mountainous areas. Using Yunnan as a case study, our research fully considered the province’s complex geographical environment and urban–rural disparity, which allowed an depiction of the travel costs and path characteristics between urban and rural areas.

As for our major findings, first, on a local scale, significant differences in URTA emerged between Guandu District and Gongshan County in Yunnan, which also reflected marked disparities in urban–rural integration levels. Guandu District features a well-developed road network, with travel times from most VSs to the county center being less than an hour, which indicates high URTA and a strong level of urban–rural integration. By contrast, most VSs in Gongshan County experience travel times exceeding 2 hours, with some exceeding 10 hours, which indicates poor URTA and underdeveloped infrastructure that restrict urban–rural integration and economic development. Despite improvements in transportation infrastructure, the URTA of Gongshan County remains limited, which highlights the critical role of transportation development in fostering regional economic and social integration. Second, at the provincial scale, URTA in Yunnan exhibited notable spatial differences. In 2015, the mean travel time from VSs to county centers was 2.13 hours, with the longest reaching 25.13 hours. By 2023, the mean travel time had decreased to 1.46 hours, with the longest at 19.28 hours, indicating an overall improvement in URTA and urban–rural integration. Central Yunnan and the areas surrounding Kunming showed higher URTA, whereas remote regions in northwestern and southern Yunnan still had low URTA, for a clear overall center–periphery pattern. Spatial autocorrelation analysis revealed high–high and low–low clustering, which further indicates significant disparities in urban–rural integration between regions. Third and last, our study has showcased new methods and indicators for evaluating and characterizing urban–rural integration. The approach adopted has valuable implications for transportation planning in mountainous rural areas globally and can serve as a reference for understanding patterns of urban–rural integration in regions with substantial geographical and spatial heterogeneity, including the Himalayas, Andes, and Alps.

## Supporting information

S1 Table Basic statistics of the number of VSs at the municipal level in Yunnan Province and the travel time to the county center. (DOCX)

S2 Table OD analysis settings. (DOCX)

S3 Table Spatial Regression Results of the Impact of Accessibility on Income (2015 & 2023). (DOCX)

S1 Fig Administrative boundaries of 16 cities and 129 counties in Yunnan. County boundaries from National Geographic Information Public Service Platform (https://www.tianditu.gov.cn/). Map Review No.: GS (2024) No. 0650. Maps created by authors using ArcGIS; no proprietary basemaps used.
(TIF)

S2 Fig Moran Index of Yunnan in 2015. Moran’s I = 0.4262 (p = 0.001), indicating significant positive spatial autocorrelation.
(TIF)

S3 Fig Moran Index of Yunnan in 2023. Moran’s I = 0.4850 (p = 0.001), indicating significant positive spatial autocorrelation.
(TIF)

S4 Fig Sensitivity index. Boxplots show changes in the sensitivity index when increasing travel speed from 5 km/h to 10 km/h and 15 km/h. The orange line indicates the median; negative values reflect reductions in travel time.
(TIF)

S1 Data S1_DATA_2015 and 2023. (CSV)

S2 Data Dictionary. (DOCX)
