# Supplementary material for: Urban–rural transportation accessibility: A novel geographical indicator for characterizing urban–rural integration
Source: PLoS One. 2026 Feb 26;21(2):e0343242. doi: 10.1371/journal.pone.0343242 (PMC12944758; doi:10.1371/journal.pone.0343242)
Supplement: S1 Table — (DOCX) [file pone.0343242.s001.docx]

**S1 Table. Basic statistics of the number of VSs at the municipal level in Yunnan Province and the travel time to the county center.**

| **Name** | **Number of VSs** | **Travel time in 2015 (hour)** | | | | **Travel time in 2023 (hour)** | | | |
| --- | --- | --- | --- | --- | --- | --- | --- | --- | --- |
|  |  | **Average** | **Maximum** | **Minimum** | **Standard Deviation** | **Average** | **Maximum** | **Minimum** | **Standard Deviation** |
| Yunnan | 144725 | 2.13 | 25.13 | 0.003 | 1.83 | 1.40 | 16.25 | 0.000003 | 1.24 |
| Kingming | 9367 | 1.51 | 9.62 | 0.003 | 1.32 | 0.98 | 6.61 | 0.000016 | 0.91 |
| Yuxi | 5000 | 1.40 | 12.04 | 0.006 | 1.31 | 0.93 | 11.28 | 0.000104 | 0.86 |
| Qujing | 14320 | 1.47 | 10.11 | 0.005 | 0.90 | 0.95 | 4.64 | 0.000111 | 0.58 |
| Zhaotong | 21835 | 2.18 | 13.74 | 0.017 | 1.61 | 1.52 | 10.87 | 0.000088 | 1.20 |
| Dali | 11555 | 2.09 | 14.50 | 0.01 | 1.84 | 1.31 | 12.93 | 0.00002 | 1.21 |
| Lijiang | 4843 | 2.77 | 15.56 | 0.01 | 2.18 | 2.07 | 15.11 | 0.000283 | 1.86 |
| Pu’er | 12027 | 2.53 | 12.34 | 0.014 | 1.79 | 1.85 | 11.71 | 0.000241 | 1.42 |
| Chuxiong | 14650 | 2.52 | 17.68 | 0.016 | 2.25 | 1.63 | 13.89 | 0.000121 | 1.50 |
| Honghe | 8875 | 1.99 | 12.35 | 0.014 | 1.70 | 1.31 | 9.23 | 0.000003 | 1.29 |
| Wenshan | 13742 | 2.08 | 14.94 | 0.016 | 1.35 | 1.32 | 9.93 | 0.000192 | 0.92 |
| Xishuangbannan | 1592 | 1.71 | 10.04 | 0.014 | 1.44 | 1.17 | 9.08 | 0.002024 | 1.10 |
| Dehong | 3079 | 1.55 | 13.51 | 0.008 | 1.37 | 0.97 | 7.10 | 0.000368 | 0.85 |
| Nujiang | 2150 | 2.94 | 15.14 | 0.016 | 2.09 | 1.89 | 12.59 | 0.003361 | 1.61 |
| Diqing | 2272 | 5.73 | 25.13 | 0.007 | 4.44 | 2.32 | 16.25 | 0.000837 | 1.99 |
| Baoshan | 9844 | 1.75 | 11.66 | 0.02 | 1.34 | 1.16 | 10.23 | 0.000924 | 0.96 |
| Lincang | 9574 | 2.50 | 13.08 | 0.013 | 1.73 | 1.57 | 9.76 | 0.002936 | 1.17 |
