# Supplementary material for: Urban–rural transportation accessibility: A novel geographical indicator for characterizing urban–rural integration
Source: PLoS One. 2026 Feb 26;21(2):e0343242. doi: 10.1371/journal.pone.0343242 (PMC12944758; doi:10.1371/journal.pone.0343242)
Supplement: S2 Table — (DOCX) [file pone.0343242.s002.docx]

**S2 Table** **OD analysis settings.**

| **Setting** | **Value / Method** |
| --- | --- |
| Software | ArcGIS 10.8 Network Analyst |
| Origins | All VSs |
| Destinations | County centers |
| Travel Cost Metric | Travel time (in hours) |
| Impedance Attribute | Speed-based time (length / speed) |
| Road Network Type | Polyline (vector-based, multilevel road network) |
| Excluded Paths | Pedestrian Paths |
