# Supplementary material for: Urban–rural transportation accessibility: A novel geographical indicator for characterizing urban–rural integration
Source: PLoS One. 2026 Feb 26;21(2):e0343242. doi: 10.1371/journal.pone.0343242 (PMC12944758; doi:10.1371/journal.pone.0343242)
Supplement: S3 Table — (DOCX) [file pone.0343242.s003.docx]

| **Variable** | **2015 OLS** | **2023 OLS** | **2015 SEM** | **2023 SEM** | **2015 SLM** | **2023 SLM** | **2015 SDM** | **2023 SDM** |
| --- | --- | --- | --- | --- | --- | --- | --- | --- |
| **Constant** | 1.216 (0.011) *** | 1.851 (0.011) *** | 1.216 (0.010) *** | 1.851 (0.010) *** | 1.172 (0.070) *** | 1.751 (0.122) *** | 1.173 (0.066) *** | 1.749 (0.118) *** |
| **Transport Accessibility Index** | 0.029 (0.013) ** | 0.018 (0.014) * | 0.031 (0.012) ** | 0.019 (0.013) * | 0.026 (0.013) * | 0.016 (0.014) * | 0.027 (0.013) ** | 0.016 (0.013) * |
| **Average Wage** | –0.010 (0.012) ns | –0.021 (0.013) * | –0.009 (0.012) ns | –0.021 (0.012) * | –0.012 (0.012) ns | –0.022 (0.012) * | –0.010 (0.012) ns | –0.024 (0.012) * |
| **Total Retail Sales** | –0.085 (0.035) ** | –0.085 (0.037) ** | –0.085 (0.034) ** | –0.084 (0.037) ** | –0.086 (0.034) ** | –0.088 (0.037) ** | –0.085 (0.034) ** | –0.087 (0.037) ** |
| **Enrollment in Secondary Schools** | –0.261 (0.018) *** | –0.341 (0.017) *** | –0.262 (0.017) *** | –0.341 (0.016) *** | –0.256 (0.019) *** | –0.333 (0.019) *** | –0.257 (0.018) *** | –0.333 (0.019) *** |
| **Primary Industry Output** | –0.004 (0.015) ns | 0.027 (0.014) * | –0.003 (0.014) ns | 0.027 (0.013) * | –0.005 (0.014) ns | 0.025 (0.013) * | –0.003 (0.014) ns | 0.027 (0.013) * |
| **Secondary Industry Output** | 0.277 (0.025) *** | 0.283 (0.021) *** | 0.278 (0.024) *** | 0.283 (0.021) *** | 0.275 (0.024) *** | 0.281 (0.021) *** | 0.276 (0.024) *** | 0.281 (0.021) *** |
| **Tertiary Industry Output** | 0.246 (0.038) *** | 0.240 (0.041) *** | 0.246 (0.037) *** | 0.240 (0.040) *** | 0.246 (0.037) *** | 0.236 (0.040) *** | 0.243 (0.037) *** | 0.235 (0.039) *** |
| **Moran’s I (Residual)** | 0.262 (p<0.001) | 0.484 p<0.001) | — | — | — | — | — | — |
| **Spatial Error Parameter (λ)** | — | — | –0.112 | –0.026 | — | — | -0.136 | –0.086 |
| **Spatial Lag Parameter (ρ)** | — | — | — | — | 0.036 | 0.054 | 0.034 | 0.054 |
| **R² or Pseudo R²** | 0.909 | 0.889 | 0.909 | 0.889 | 0.909 | 0.889 | 0.909 | 0.889 |

**S3 Table. Spatial Regression Results of the Impact of Accessibility on Income (2015 & 2023)**

Table Note: Coefficients with standard errors in parentheses. Dependent variable: log per capita income. Higher accessibility index = better access. λ, ρ, θ are spatial parameters. Adjusted R² (OLS) and pseudo R² (spatial models). Global Moran’s I tests OLS residual autocorrelation. Significance: *** p < 0.01; ** p < 0.05; * p < 0.10; n.s. = not significant.
