# Supplementary figures and images for: Urban–rural transportation accessibility: A novel geographical indicator for characterizing urban–rural integration
Source: PLoS One. 2026 Feb 26;21(2):e0343242. doi: 10.1371/journal.pone.0343242 (PMC12944758; doi:10.1371/journal.pone.0343242)

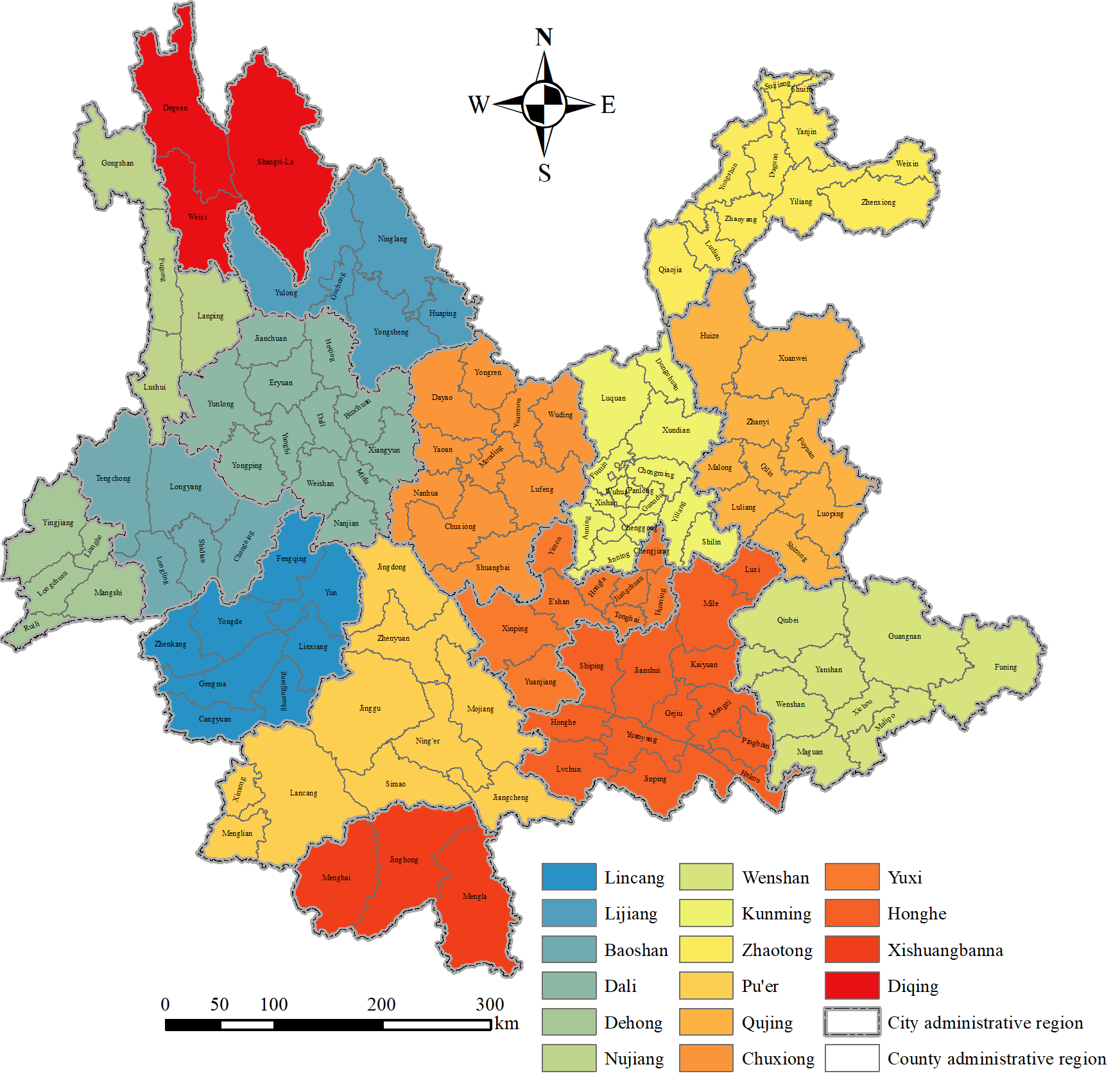

Supplement: S1 Fig — County boundaries from National Geographic Information Public Service Platform (https://www.tianditu.gov.cn/). Map Review No.: GS (2024) No. 0650. Maps created by authors using ArcGIS; no proprietary basemaps used. (TIF) [file pone.0343242.s004.tif]

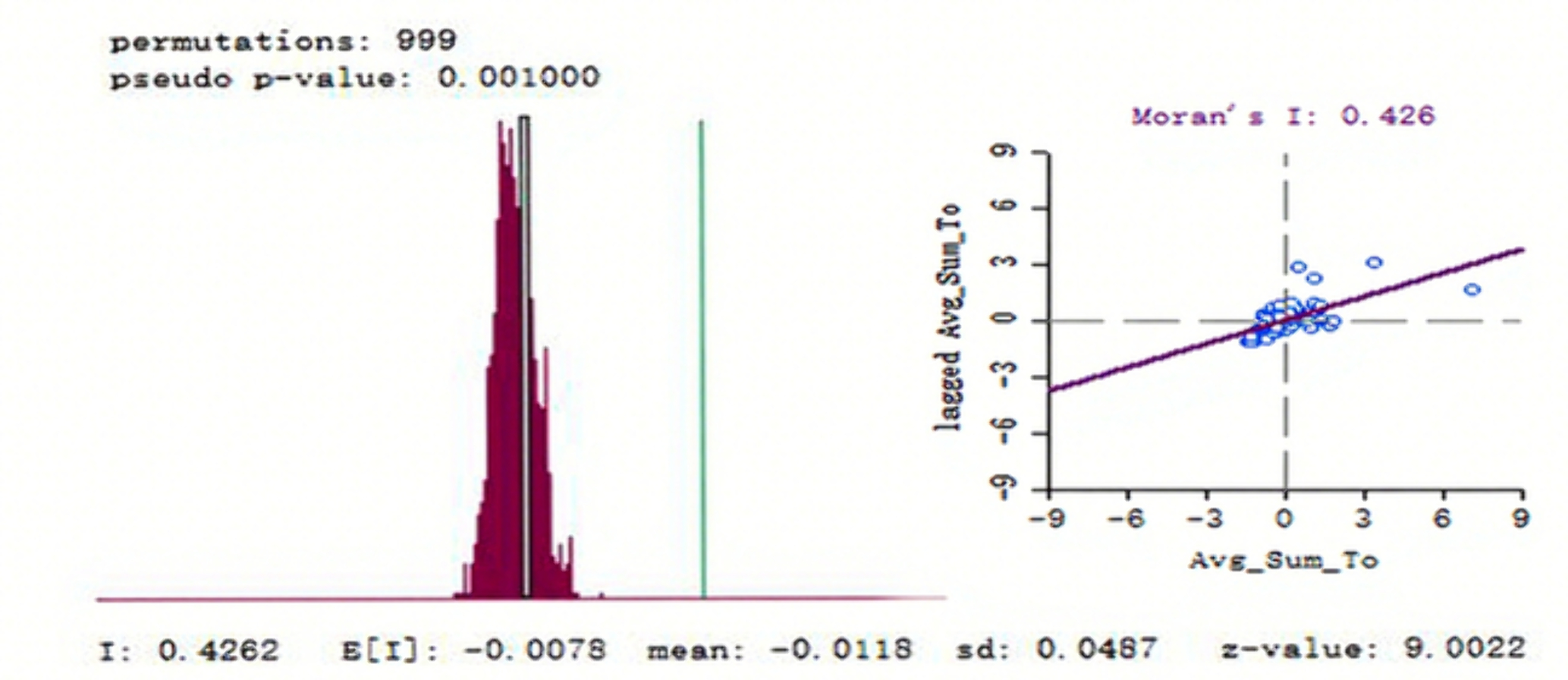

Supplement: S2 Fig — Moran’s I = 0.4262 (p = 0.001), indicating significant positive spatial autocorrelation. (TIF) [file pone.0343242.s005.tif]

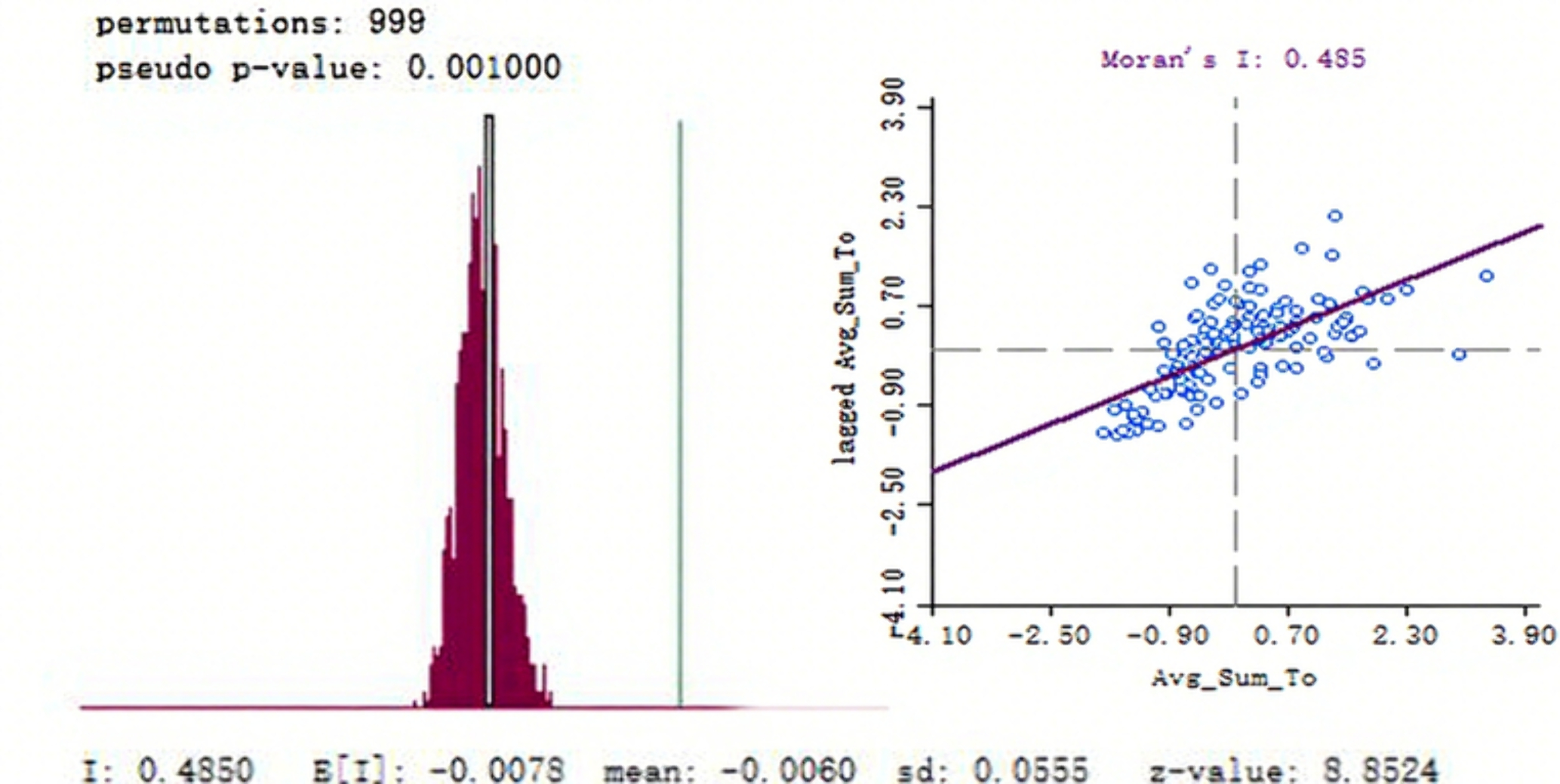

Supplement: S3 Fig — Moran’s I = 0.4850 (p = 0.001), indicating significant positive spatial autocorrelation. (TIF) [file pone.0343242.s006.tif]

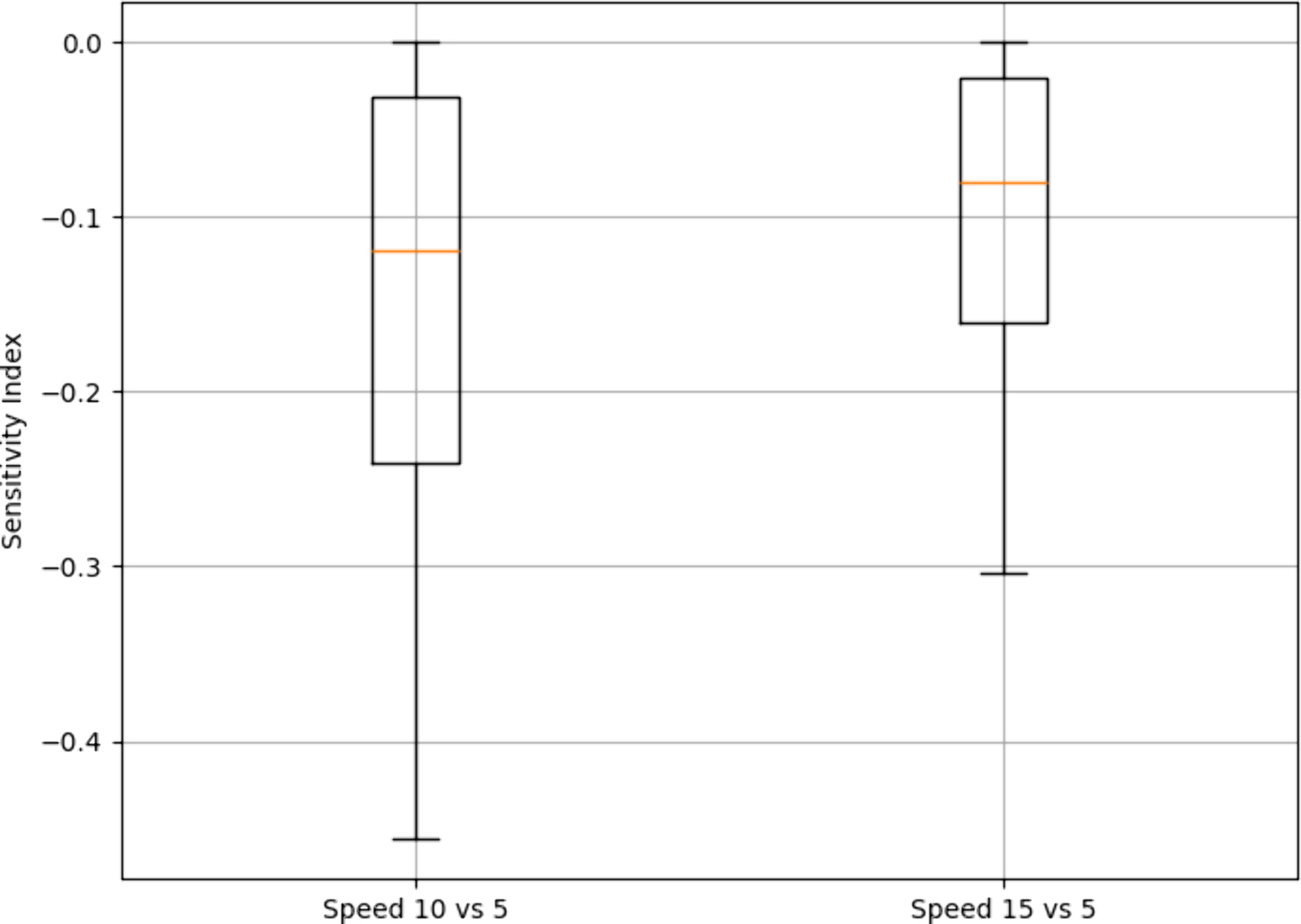

Supplement: S4 Fig — Boxplots show changes in the sensitivity index when increasing travel speed from 5 km/h to 10 km/h and 15 km/h. The orange line indicates the median; negative values reflect reductions in travel time. (TIF) [file pone.0343242.s007.tif]
