# Supplementary material for: Urban–rural transportation accessibility: A novel geographical indicator for characterizing urban–rural integration
Source: PLoS One. 2026 Feb 26;21(2):e0343242. doi: 10.1371/journal.pone.0343242 (PMC12944758; doi:10.1371/journal.pone.0343242)
Supplement: S2 Data — (DOCX) [file pone.0343242.s009.docx]

**Data Dictionary**

The dataset S1_DATA_2015 and 2023.csv contains village-level travel time information describing urban–rural transport accessibility in the study area for the years 2015 and 2023. Each record corresponds to a village settlement (VS), representing a rural residential location included in the analysis.

The dataset integrates spatial location information and network-based travel time estimates, and is intended to support reproducibility and transparency of the analyses presented in the study.

**Data generation method**

Travel time values were derived using road network–based analysis. For each village settlement, the shortest travel time to its corresponding county-level administrative center was calculated using a geographic information system (GIS) network analysis approach. Travel time estimation was based on road-class-specific maximum speed limits and reflects the fastest travel time under standard travel conditions. The same methodology was applied consistently for both 2015 and 2023 to ensure comparability across years.

**Variables and definitions**

The dataset includes the following variables:

VS ID

A unique numeric identifier for each village settlement (VS). This identifier is used to distinguish individual village records within the dataset.

Lon (°)

Longitude of the VS, expressed in decimal degrees. Coordinates are provided in the WGS 84 geographic coordinate system.

Lat (°)

Latitude of the VS, expressed in decimal degrees. Coordinates are provided in the WGS 84 geographic coordinate system.

2015_Travel Time (h)

The shortest travel time from the village settlement to its associated county-level administrative center in 2015. Values are expressed in hours (h).

2023_Travel Time (h)

The shortest travel time from the village settlement to its associated county-level administrative center in 2023. Values are expressed in hours (h).

**Data format and size**

The dataset is provided in comma-separated values (CSV) format, which can be directly opened and processed using common data analysis and GIS software. The file size is approximately 8.83 MB.

**Notes on data use**

All variables are provided at the village settlement level. Travel time values are continuous numerical variables and are directly readable without additional transformation. Geographic coordinates allow the dataset to be linked with spatial layers or mapped for further spatial analysis.
